# Prevalence and risk factors for burnout among rheumatologists: a systematic review and meta-analysis

**DOI:** 10.1007/s00296-026-06275-1

**Published:** 2026-08-25

**Authors:** Zaneta N. Harlianto, Netanja I. Harlianto

**Affiliations:** 1 Faculty of Medicine, Anton de Kom Universiteit Suriname, Paramaribo, Suriname; 2 Utrecht University, Utrecht, the Netherlands; 3 University Medical Center Utrecht, Utrecht, the Netherlands

**Keywords:** Burnout, Stress disorders, Rheumatology, Rheumatologists, Workload, Prevalence

## Abstract

The aim of this study was to systematically evaluate the prevalence and risk factors of burnout among rheumatologists. Medline and EMBASE were queried from inception until June 2026 for studies on burnout prevalence and/or risk factors in rheumatologists. Pooled prevalence was estimated with random effects meta-analysis, and risk factors for burnout were qualitatively assessed. A total of 12 studies were included, of which 7 prevalence studies (n=1342) were meta-analyzed that assessed burnout with the validated Maslach Burnout Inventory. The pooled prevalence of burnout was 51% (95%CI:39%-63%), emotional exhaustion 26% (95%CI: 18%-34%), depersonalization 20% (95%CI: 13%-28%), and low sense of personal accomplishment 31% (95%CI: 14%-51%). Findings were characterized by significant heterogeneity. Publication bias and subgroup analyses could not be performed. Individual risk factors for burnout included younger age, comorbidities, depression, lack of exercise, shorter years of practice/experience, and lower income in Latin America and the Middle East/North Africa. Work factors associated with burnout included longer work hours, practice types other than group practice, receiving phone calls from patients, being dissatisfied with the specialty, work, and rheumatology practice. More than half of rheumatologists have reported at least one dimension of burnout (emotional exhaustion, depersonalization, and personal accomplishment). These findings emphasize the need to address burnout and its risk factors in rheumatologists. Institutions should prioritize strategies aimed at reducing workplace stressors and promoting rheumatologist well-being.

**Supplementary Information:**

The online version contains supplementary material available at https://doi.org/10.1007/s00296-026-06275-1.

## Introduction

Burnout among physicians has emerged as a major concern in healthcare, affecting various medical specialties in medicine. Depending on the definition and cutoff used, the reported prevalence of burnout among physicians is between 0 and 90% [1]. Burnout is characterized by work-related stress with emotional exhaustion, depersonalization, and/or feeling low personal accomplishment [2]. Emotional exhaustion reflects the strain of continuous clinical workload and administrative demands; depersonalization is characterized by a detachment from work and emotional distancing from colleagues and patients; and a low sense of personal accomplishment may reflect the perceived decline in professional efficacy and a lack of professional achievement. One of the most used tools to assess burnout includes the Maslach Burnout Inventory (MBI), a standardized and validated questionnaire that explores burnout and its three subdimensions [3]. The MBI has been extensively applied in physician populations and remains the reference standard in burnout research and the clinical setting [1, 3]. Burnout in physicians has been associated with increased risk for medical errors [4] and adverse patient outcomes [5].

There has been a growing interest for burnout in rheumatologists, reflected by the growing recent number of publications on this topic [6–9]. In rheumatology, clinicians manage chronic, complex autoimmune diseases requiring sustained patient relationships and long-term therapeutic decision-making. These factors in combination with personal and work-related factors, may make rheumatologists more susceptible to burnout.

To the best of our knowledge, no prior study has systematically investigated the pooled prevalence of burnout in rheumatologists and its associated risk factors. These findings are important to understand the growing challenges facing the rheumatology workforce, and for informing interventions aimed at improving physician well-being, reducing attrition, and maintaining high-quality patient care. Thus, this study aimed to systematically review the evidence of published studies on burnout prevalence and to explore the reported risk factors associated with burnout in rheumatologists.

## Methods

### Search strategy

This systematic review adhered to the Preferred Reporting Items for Systematic Reviews and Meta-Analyses (PRISMA) guidelines [10]. A systematic literature search of Medline and EMBASE [11] was performed and updated on June 8th, 2026 to identify articles reporting burnout in rheumatology physicians. A search was performed with the syntax “burnout” AND “rheumatologists” and relevant synonyms (Full search listed in Supplementary Table S1). Title and abstract screening were performed by two reviewers, with discrepancies of screening discussed by consensus. No language restrictions were applied, and cross-referencing was employed to identify additional articles not included in the electronic search.

### Article selection and data extraction

Inclusion criteria were studies reporting burnout in rheumatology physicians (residents and/or specialists) and provided data on either prevalence and/or risk factors of burnout. We excluded studies that did not investigate burnout, and investigations solely reporting the effectiveness of measures to reduce burnout. Data extraction was performed by two reviewers, with discrepancies discussed by consensus. Extracted data included author, year of publication, sampling period, response rate, type of practitioner, mean age, percentage of males, burnout assessment tool, definition of burnout, and factors associated with burnout. The JBI Critical appraisal tool for prevalence/incidence systematic reviews was used for quality assessment of included studies [12].

### Data analysis

Random effects meta-analysis was employed to calculate the pooled prevalence of burnout, emotional exhaustion, depersonalization, and low sense of personal accomplishment. To reduce heterogeneity, only studies that evaluated burnout using the MBI tool were meta-analyzed. We performed a Freeman–Tukey double arcsine square root transformation to stabilize the variance of raw proportions [13]. The extent of statistical heterogeneity was evaluated using Higgin’s and Thompson’s I^2^ [14], which was stratified as low (< 25%), moderate (25–50%), and high (> 50%). The remaining studies were qualitatively described, including risk factors and protective factors associated with burnout. Subgroup analyses were not performed due to limited number of studies. Instead, leave-one-out sensitivity analyses were conducted to assess the robustness of the pooled estimates and identify any influential studies. Publication bias was not performed because the number of pooled studies was less than 10. Analyses were performed using R Statistical Software version 4.5.2. (R Foundation for Statistical Computing, Vienna, Austria) using the meta and metafor package. A *p*-value of < 0.05 was considered statistically significant.

## Results

### Study selection and characteristics

**Fig. 1:**
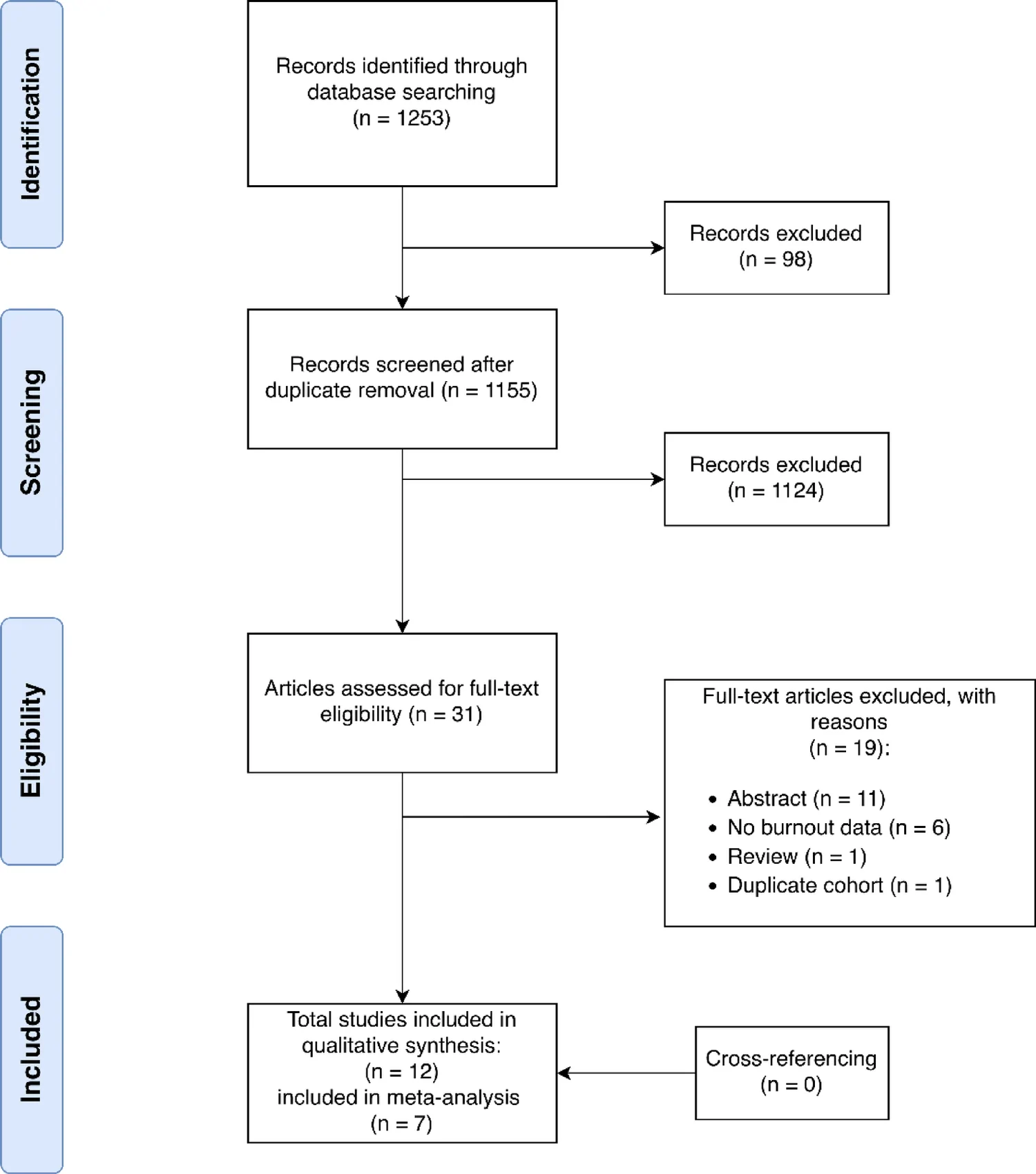
Flowchart of study selection

A total of 1155 articles were identified after removing duplicates and screened for inclusion. Hereafter, full-text evaluation was performed for 42 articles, of which 30 were excluded with reason. Ultimately, 12 studies were included in this systematic review (Fig. 1) [6–9, 15–22], describing a total of 2132 physicians in the field of rheumatology (median sample size: 128; IQR: 103–240).

The included studies were conducted across diverse geographic regions, including North America, Asia, Europe, the Middle East, and Latin America. Most studies included rheumatologists (*n* = 6; 50.0%), followed by residents (*n* = 2; 16.7%), fellows (n = 1; 8.3%), pediatric rheumatologists (*n* = 1; 8.3%), (pediatric) rheumatologists and fellows (*n* = 1; 8.3%), rheumatologists and residents (*n* = 1; 8.3%), and rheumatologists and pediatric rheumatologists (*n* = 1; 8.3%). Overall, rheumatologists were involved either alone or in combination with other groups in 9 of 12 studies (75.0%). Study specific quality assessment is provided in Supplementary Table S2.

### Prevalence of burnout

**Fig. 2:**
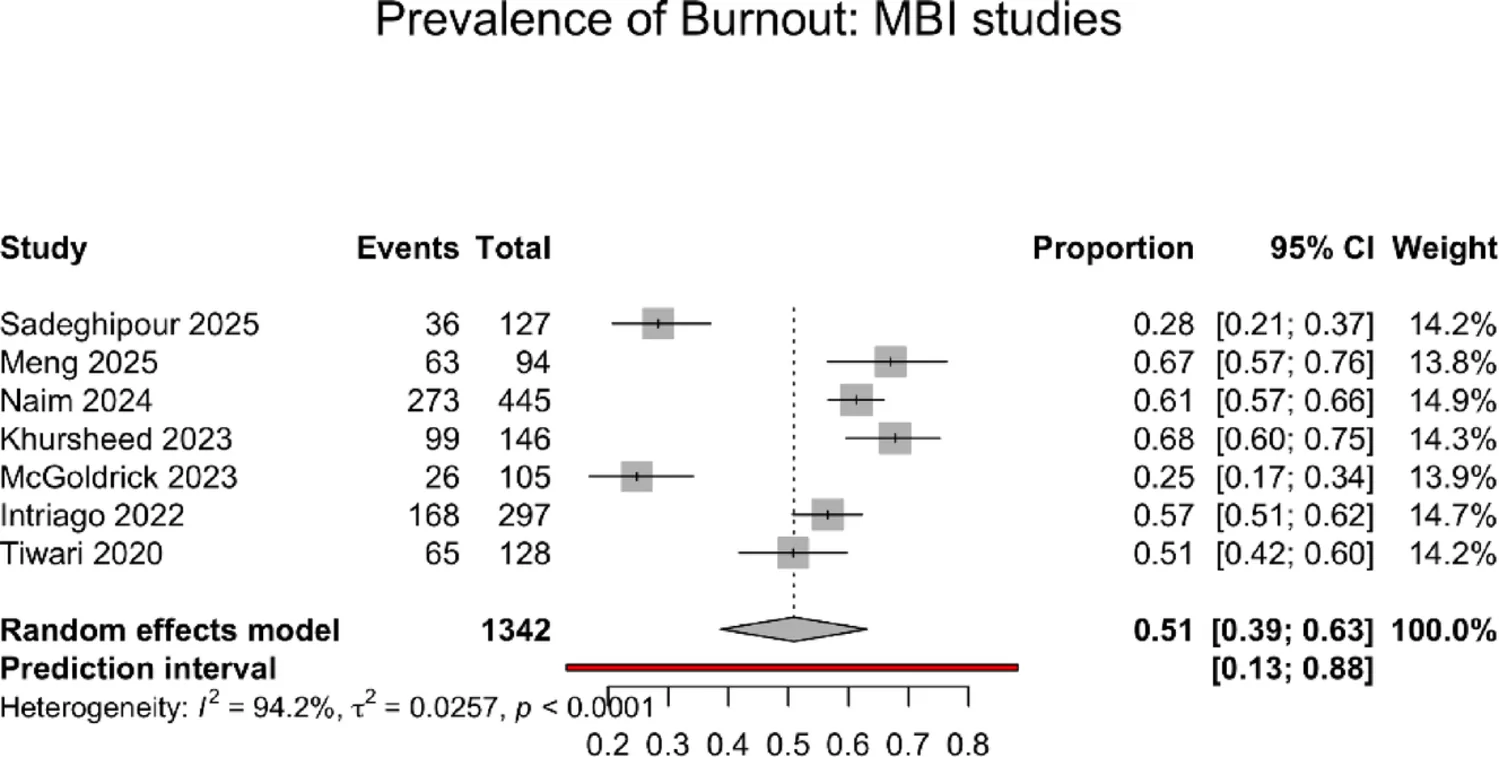
Pooled prevalence of burnout

Burnout assessment was performed using the full MBI in seven studies, questionnaires derived from MBI subscales in three studies, and non-validated burnout questionnaires in two studies. For seven studies that used the MBI (*n* = 1342), the pooled prevalence of burnout was 51% (95%CI: 39%−63%), I^2^: 94% (95%CI: 90%−97%) (Fig. 2**)**. The pooled prevalence was similar at 54% (95%CI: 41%−67%), when excluding the two studies with fellows only, and rheumatologists and fellows. Excluding the largest study of Naim et al., gave a pooled burnout prevalence of 49% (95%CI: 35%−63%) Table 1.

**Table 1:** Study characteristics

Study	Region	Period	Participants	Response rate	Sample size	Mean age	% male	Burnout assessment
Sadeghipour 2025	Iran	NS	Rheumatologists	53%	127	NS	35	MBI
Meng 2025	China	NS	Rheumatologists	94%	94	39	35	MBI
Naim 2024	Middle East and North Africa	Spring 2022	Rheumatologists and fellows	14.2%	445	45	62	MBI
Chandwar 2023	India	NS	Residents	NS	78	31	70	Non-validated likert
Khursheed 2023	South Asia	12–2021 to 04–2022	Rheumatologists	NS	146	39*	49	MBI
Kozu 2023	Latin America	07–2020 to 09–2020	Pediatric rheumatologists	40%	126	44	12	Single-item MBI
Kuhlmann 2023	Germany	01–2021 to 02–2021	Rheumatologists and residents	NS	101	NS	58	Non-validated likert
McGoldrick 2023	USA	01–2019 to 02–2019	Fellows	18%	105	NS	35	MBI
Intriago 2022	Latin America	08–2020 to 11–2020	Rheumatologists	NS	297	48	38	MBI
Kulhawy-Wibe 2022	Canada	10–2020 to 03–2021	Rheumatologists and pediatric rheumatologists	44%	183	47*	38	Mini-z
Young 2022	Worldwide	08–2020 to 10–2020	Residents	NS	302	NS	NS	Two-item MBI
Tiwari 2020	USA	02–2019	Rheumatologists	80%	128	NS	46	MBI

*median age. *NS* Not specified; *MBI* Maslach Burnout Inventory

**Fig. 3:**
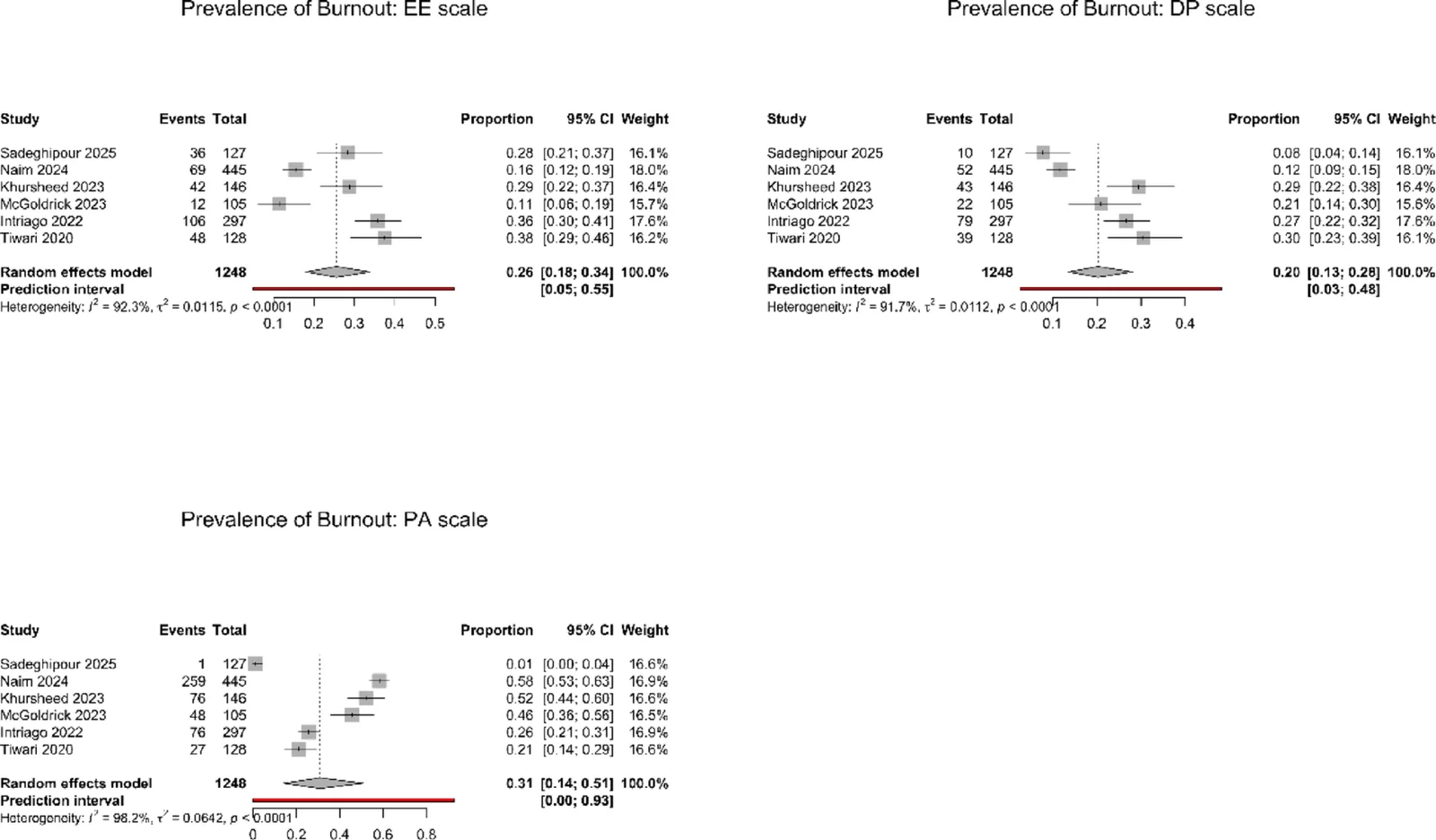
Pooled prevalence of burnout subdimensions. Emotional exhaustion (**A**), depersonalization (**B**), and low sense of personal accomplishment (**C**). *EE* Emotional exhaustion; *DP* Depersonalization; *PA* Personal accomplishment

Six studies reported the MBI subscales of emotional exhaustion, depersonalization, and a low sense of personal accomplishment. The proportion of emotional exhaustion was 26% (95%CI: 18%−34%), I^2^: 92% (95%CI: 86%−96%) (Fig. 3A), the proportion of depersonalization was 20% (95%CI: 13%−28%), I^2^: 92% (95%CI: 85%−96%) (Fig. 3B), and the proportion with a low sense of personal accomplishment was 31% (95%CI: 14%−51%), I^2^: 98% (95%CI: 97%−99%) (Fig. 3C). Publication bias was not formally assessed because only seven studies were included.

### Risk factors for burnout

Seven studies reported risk factors associated with burnout presence and burnout subscales **(**Table 2**)**. Individual factors associated with burnout included younger age [6–8, 14, 16], comorbidities (adjusted OR 2.29, 95%CI: 1.05–5.02) [9], lack of exercise (adjusted OR 5.00, 95% CI 1.3–18.5) [16] and depression (adjusted OR 4.11, 95%CI: 1.94–8.70) [9]. Female sex was associated with burnout in one study [16], whereas four other studies found no association with sex [7, 8, 15, 16]. Lower income was associated with burnout in Latin America and the Middle East/North Africa [8, 9], whereas income was not associated with burnout in the USA [16]. Longer years of practice/experience was negatively associated with burnout in three studies [6, 8, 15], whereas Tiwari et al. [16] found no association between years of experience and burnout.

Work factors associated with burnout included working more than 60 h per week (OR 2.6, 95% CI 1.16–5.6) [16]; practitioners in group practice (OR 0.43, 95% CI 0.20–0.92) [16], receiving phone calls from patients (OR 2.51, 95%CI: 1.15–2.97) [8], infusion centre at rheumatology practice (OR 0.59, 95%CI: 0.37–0.93) [8], unhappiness with the electronic health record (OR 2.86, 95% CI 1.23–6.65) [16]. Regarding satisfaction, three studies found being unsatisfied with the specialty (OR 2.1, 95%CI: 1.3–3.4) [8], spending > 20% of time on satisfying work (OR 0.32, 95% CI 0.15–0.71 [16], and satisfaction with their practice as rheumatologists (OR 0.60, 95%CI: 0.42–0.85) [9] were associated with burnout.

**Table 2:** Prevalence and risk factors associated with burnout

Study	Burnout assessment	Burnout definition	Burnout prevalence	EE prevalence	DP prevalence	PA prevalence	Risk factor for burnout	Protective factor for burnout
Sadeghipour 2025	MBI	NS	33.9%	28.3%	10.0%	0.6%	Having children correlated negatively with PA (*r* = − 0.201, *p* = 0.024).	“Age correlated negatively with EE (ρ = − 0.311, *p* < 0.001), DP (ρ = − 0.192, *p* = 0.030), and PA (ρ = − 0.370, *p* < 0.001). Medical experience was negatively associated with EE (ρ = − 0.275, *p* = 0.002) and PA (ρ = − 0.310, *p* < 0.001). Single rheumatologists scored higher in PA (mean = 22.37 vs. 17.16; *p* = 0.002).”
Meng 2025	MBI	NS	67%	NS	NS	NS	“The high workload was associated with high EE and DP scores (*P* < 0.001)”	“Age and years of work were found to have a negative correlation with burnout PA scores (*P* < 0.01). management support and intrinsic motivation were negatively related to burnout risk (*P* < 0.001).”
Naim 2024	MBI	EE scores of ≥ 27, DP scores of ≥ 10, and PA scores of ≤ 33 are considered positive for burnout	61.3%	15.6%	11.6%	58.1%	“Younger age (OR 2.1, 95%CI: 1.4–3.3), lower monthly income (OR 3.3, 95%CI: 1.5–7.6), specialty dissatisfaction (OR 2.1, 95%CI: 1.3–3.4), receiving phone calls from patients (OR 2.51, 95%CI: 1.15–2.97)”	“longer practice duration (OR 0.97, 95%CI: 0.95–0.99); infusion centre at rheumatology practice (OR 0.59, 95%CI: 0.37–0.93)”
Chandwar 2023	Non-validated likert	NS	79%	-	-	-	-	-
Khursheed 2023	MBI	EE scores of ≥ 27, DP scores of ≥ 10, and PA scores of ≤ 33 are considered positive for burnout	67.8%	28.8%	29.5%	52.1%	-	-
Kozu 2023	Single-item MBI	Modified EE score ≥ 3 out of 5	77.0%	-	-	-	-	-
Kuhlmann 2023	Non-validated likert	NS	32.0%	-	-	-	-	-
McGoldrick 2023	MBI	EE scores > 27, DP scores > 10*	24.7%	11.4%	21.0%	45.7%	-	“Older age (OR: 0.34, 95%CI: 0.12–1.00)”
Intriago 2022	MBI	EE scores of ≥ 27, DP scores of ≥ 10, and PA scores of ≤ 33 are considered positive for burnout	56.6%	35.7%	26.6%	25.6%	“comorbidities (OR 2.29, 95%CI: 1.05–5.02) and depression (OR 4.11, 95%CI: 1.94–8.70)”	“Satisfaction with their practice as rheumatologists (OR 0.60, 95%CI: 0.42–0.85) and with their income (OR 0.75, 95%CI: 0.58–0.98)”
Kulhawy-Wibe 2022	Single-item burnout Mini-X	scores of 1–3 out of 5	51.0%	-	-	-	“female sex (OR 2.42, 95% CI 1.07–5.60)”	“older age (OR 0.95, 95% CI 0.92–0.99)”
Young 2022	Two-item MBI	4–5 out of 5	50.0%	-	-	-	-	-
Tiwari 2020	MBI	EE scores of ≥ 27, DP scores of ≥ 10, and PA scores of ≤ 33 are considered positive for burnout	50.8%	37.5%	30.5%	21.6%	“Unhappiness with EHR (OR 2.86, 95% CI 1.23–6.65); lack of exercise (OR 5.00, 95% CI 1.3–18.5). Work hours > 60/week (OR 2.6, 95% CI 1.16–5.6)”	“Practitioners in group practice (OR 0.43, 95% CI 0.20–0.92). Practitioners who spend > 20% of their time of their time in personally satisfying work (OR 0.32, 95% CI 0.15–0.71).”

*PA scores not considered. *EE* Emotional exhaustion, *DP* Depersonalization, *PA* Personal accomplishment, *OR* adjusted odds ratio; 95%CI: 95% confidence interval

## Discussion

This systematic review and meta-analysis aimed to assess burnout prevalence and its risk factors in physicians working in the field of rheumatology. We found that the pooled prevalence of burnout was 51% (95%CI: 39%−63%), meaning more than half of rheumatologists reported at least one dimension of burnout. A low sense of personal accomplishment was most frequent at 31% (95%CI: 14%−51%), followed by emotional exhaustion 26% (95%CI: 28%−34%), and depersonalization 20% (95%CI: 13%−28%). The prevalence of burnout in our study is in line with estimates from other specialties, including surgical disciplines and internal medicine subspecialties [1, 23, 24]. These findings indicate that burnout and its subsymptoms are common among rheumatologists worldwide and are important to inform organizational strategies and targeted interventions aimed at improving rheumatologist well-being. A portion of studies in our review used data collected during the Covid-19 pandemic. However, we also included published studies that collected data in 2019 or from 2023 onwards, with similar or higher prevalences of burnout. For example, Tiwari et al. [16] found a burnout prevalence of 50.8%, using data from 2019. Likewise, McGolddrick [7] used data from 2019 and found a prevalence of 24.7%, but they did not include decreased PA scores in their classification of burnout, which resulted in a lower prevalence. Finally, Meng et al. [15] found a moderate risk of burnout in 67% of their study population. The wide prediction interval (19%–86%) further highlights the substantial between-study heterogeneity observed in this review. Although the pooled prevalence provides an estimate of the average burnout burden, burnout prevalence is likely to vary considerably across different healthcare systems, practice settings, and physician populations. Consequently, the pooled estimate should be interpreted with caution and should not be assumed to represent all clinical contexts.

Our qualitative results identified various risk factors and protective factors associated with burnout. Most studies found no association between burnout and sex. However, younger age and less years of practice were found to be associated with burnout, alongside other personal factors such as comorbidities and depression. It has been well established that a higher workload is associated with burnout, which was reflected in the risk factors of more working hours per week, and additional work through the EHR or by taking direct phone calls from patients. Other factors associated with burnout from the literature include organizational factors such as extensive documentation, a lack of time for task completion and insufficient work diversity [25]. A prior study evaluated which factors are associated with work satisfaction in rheumatology. The authors found that better reimbursement and less administrative work were associated with work satisfaction [26]. Satisfaction with work has been identified as a protective factor against work-related stress [27]. The observed associations with country income level and geographic region should be interpreted cautiously, as these variables are closely related and may confound one another. Differences in healthcare infrastructure, physician workforce, organizational support, and socioeconomic conditions across regions may contribute to the observed variation in burnout prevalence, and the independent effects of these factors could not be disentangled with the available data.

A recent qualitative study explored themes related to burnout in early-career rheumatologists [28]. The authors interviewed 64 rheumatologists and found that burnout among rheumatologists may be driven by specialty-specific factors, including high administrative burdens related to biologic therapies, such as prior authorizations and insurer-mandated peer-to-peer reviews. Moreover, rheumatologists may experience frustration from perceived inadequate compensation for the complex cognitive work required for increasingly sophisticated diagnostic and therapeutic decision-making [28]. Most studies included in this review employed a cross-sectional design. Therefore, the identified factors should be interpreted as correlates rather than causal determinants of physician burnout, as the temporal relationship between exposures and burnout cannot be established.

Our results are important for rheumatology workforce planning and for the evaluation of interventions with the aim to improve physician well-being, reducing attrition, and maintaining high-quality patient care in rheumatology [29]. Staffing shortages may contribute substantially to burnout among rheumatologists by increasing clinical workload, reducing opportunities for recovery, and placing additional administrative and patient-care responsibilities on existing physicians [29]. Rheumatology workforce projections have estimated a shortage of rheumatologists in 2030, despite the growth in the number of rheumatologists [30, 31]. Herein, interventions for burnout improvement are important to address and reduce burnout in rheumatologists. A recent systematic review evaluated the published evidence and identified potential interventions to reduce burnout in rheumatology [32]. Although the authors found no intervention studies conducted among rheumatology populations, they described potential strategies to address burnout. These included workflow and organizational strategies; peer support and formal communication training; leadership support, addressing stress, mental health, and mindfulness. Of all interventions, the highest quality of evidence was for mindfulness [32]. A recent meta-analysis of randomized studies identified personal coaching, but not mindfulness, as an effective intervention to reduce burnout in physicians [33]. Effective interventions require a multifaceted approach at multiple levels, usually at the organizational level [28]. Given the substantial prevalence of burnout observed, additional research is warranted to develop and assess evidence-based interventions to improve the wellbeing of rheumatologists, prevent burnout, and promote workforce sustainability.

The limitations of our study should be mentioned. Our results were characterized by high heterogeneity, which could be due to different possible factors including participant characteristics, healthcare systems, and work workplace environments. These factors could not be explored in subgroup analyses due to the limited number of studies. Hence, the estimates of our study should therefore be interpreted with caution. The findings should also be interpreted considering several factors that may have contributed to heterogeneity across studies. Response rates varied considerably, raising the possibility of non-response bias if physicians experiencing higher or lower levels of burnout were differentially represented. The methodological quality of the included studies also varied, which may have influenced the reliability of individual prevalence estimates. In addition, some studies were conducted during the COVID-19 pandemic, a period characterized by substantial disruptions to healthcare delivery and increased occupational stress, potentially affecting reported burnout prevalence. Moreover, differences in healthcare systems, working conditions, organizational structures, and physician roles across countries may have contributed to variations in burnout estimates and limit the direct comparability of findings. Finally, as the number of included studies was less than 10, publication bias could not be formally assessed. Nevertheless, we have reviewed the largest collection of studies reporting burnout prevalence in rheumatologists and have provided qualitative information on protective factors and risk factors for burnout.

In conclusion, this systematic review and meta-analysis found that more than half of rheumatologists have reported at least one dimension of burnout (emotional exhaustion, depersonalization, and personal accomplishment). These findings underscore the need to address burnout and its risk factors within this population. Institutions should prioritize strategies aimed at reducing workplace stressors and promoting rheumatologist well-being. Given the variability in burnout across settings and physician characteristics, interventions should be tailored to local healthcare contexts. Further longitudinal studies are needed on optimal management and potential interventions to prevent burnout in rheumatologists, and to better understand temporal trends and identify modifiable factors.

## Supplementary Information

Below is the link to the electronic supplementary material.

Supplementary Material 1

## Data Availability

Data is available from the corresponding author upon reasonable request.
