# Supplementary material for: Prevalence and risk factors for burnout among rheumatologists: a systematic review and meta-analysis
Source: Rheumatol Int. 2026 Aug 25;46(9):245. doi: 10.1007/s00296-026-06275-1 (PMC13506508; doi:10.1007/s00296-026-06275-1)
Supplement: Supplementary file 1 [file 296_2026_6275_MOESM1_ESM.docx]

**Supplemental material**

Supplementary Table S1.

|  | **Medline** | **EMBASE** |
| --- | --- | --- |
| #1 | (burnout OR “burned out” OR depersonalization OR "emotional exhaustion" OR burnout, professional [MESH] or emotional stress [MESH] or psychological stress [MESH] or stress, psychological [MESH] OR compassion fatigue [MESH]) | burnout:ti,ab,kw OR  "burned out":ti,ab,kw OR  depersonalization:ti,ab,kw OR  "emotional exhaustion":ti,ab,kw OR  'burnout'/exp OR  'emotional stress'/exp OR  'psychological stress'/exp OR  'compassion fatigue'/exp |
| #2 | rheumatology OR rheumatologist OR rheumatologists[MeSH] OR "attending rheumatologist" OR “rheumatology residents” OR “rheumatology fellow” OR “rheumatology attending” OR “rheumatology consultant” | 'rheumatology'/exp OR  'rheumatologist'/exp OR  rheumatology:ti,ab,kw OR  rheumatologist*:ti,ab,kw OR  "attending rheumatologist":ti,ab,kw OR  "rheumatology resident*":ti,ab,kw OR  "rheumatology fellow*":ti,ab,kw OR  "rheumatology attending*":ti,ab,kw OR  "rheumatology consultant*":ti,ab,kw |
| Final search | # 1 AND #2 | # 1 AND #2 |

Supplementary Table S2. Individual quality assessment

| **Author** | **Q1** | **Q2** | **Q3** | **Q4** | **Q5** | **Q6** | **Q7** | **Q8** | **Q9** |
| --- | --- | --- | --- | --- | --- | --- | --- | --- | --- |
|  | **Inclusion** | **Sampling** | **Sample size** | **Setting** | **Coverage** | **Identification** | **Reliability** | **Analysis** | **Response rate** |
| Sadeghipour 2025 | yes | no | yes | yes | yes | yes | yes | NR | no |
| Meng 2025 | yes | no | no | yes | yes | yes | yes | NR | yes |
| Naim 2024 | yes | no | yes | yes | yes | yes | yes | yes | no |
| Chandwar 2023 | yes | no | no | yes | yes | no | yes | yes | NR |
| Khursheed 2023 | yes | no | yes | yes | yes | yes | yes | yes | NR |
| Kozu 2023 | yes | no | yes | yes | yes | no | yes | yes | no |
| Kuhlman 2023 | yes | no | yes | yes | yes | no | yes | yes | NR |
| McGoldrick 2023 | yes | no | yes | yes | yes | yes | yes | yes | no |
| Intriago 2022 | yes | no | yes | yes | yes | yes | yes | yes | NR |
| Kulhawy-Wibe 2022 | yes | no | yes | yes | yes | no | yes | yes | no |
| Young 2022 | yes | no | yes | yes | yes | no | yes | yes | NR |
| Tiwari 2020 | yes | no | yes | yes | yes | yes | yes | yes | yes |

NR: not reported
